# Outcomes of surgery and subsequent therapy for central nervous system oligoprogression in EGFR-mutated NSCLC patients

**DOI:** 10.1186/s12957-023-03248-7

**Published:** 2023-11-25

**Authors:** Pang-Shuo Perng, Heng-Juei Hsu, Jung-Shun Lee, Liang-Chao Wang, Chih-Yuan Huang, Chih-Hao Tien, Yu-Hsuan Lai, Po-Lan Su, Hao-Hsiang Hsu, Liang-Yi Chen, Po-Hsuan Lee

**Affiliations:** 1 Section of Neurosurgery, Department of Surgery, National Cheng Kung University Hospital, College of Medicine, National Cheng Kung University, Tainan, Taiwan; 2 Department of Surgery, Tainan Municipal Hospital, Tainan, Taiwan; 3 Department of Cell Biology and Anatomy, College of Medicine, National Cheng Kung University, Tainan, Taiwan; 4 Institute of Basic Medical Sciences, College of Medicine, National Cheng Kung University, Tainan, Taiwan; 5 Department of Oncology, National Cheng Kung University Hospital, College of Medicine, National Cheng Kung University, Tainan, Taiwan; 6 Institute of Clinical Medicine, College of Medicine, National Cheng Kung University, Tainan, Taiwan; 7 Department of Internal Medicine, National Cheng Kung University Hospital, College of Medicine, National Cheng Kung University, Tainan, Taiwan

**Keywords:** Non-small cell lung cancer, Oligoprogression, Oligometastasis, Tyrosine kinase inhibitor, Metastasis, Metastatic brain tumors

## Abstract

**Background:**

Oligoprogression is an emerging issue in patients with epidermal growth factor receptor (EGFR)-mutated non-small cell lung cancer (NSCLC). However, the surgical treatment for central nervous system (CNS) oligoprogression is not widely discussed. We investigated the outcomes of craniotomy with adjuvant whole-brain radiotherapy (WBRT) and subsequent therapies for CNS oligoprogression in patients with EGFR-mutated NSCLC.

**Methods:**

NSCLC patients with CNS oligoprogression were identified from a tertiary medical center. The outcomes of surgery with adjuvant WBRT or WBRT alone were analyzed, along with other variables. Overall survival and progression-free survival were analyzed using the log-rank test as the primary and secondary endpoints. A COX regression model was used to identify the possible prognostic factors.

**Results:**

Thirty-seven patients with CNS oligoprogression who underwent surgery or WBRT were included in the study after reviewing 728 patients. Twenty-one patients underwent surgery with adjuvant WBRT, and 16 received WBRT alone. The median overall survival for surgery and WBRT alone groups was 43 (95% CI 17–69) and 22 (95% CI 15–29) months, respectively. Female sex was a positive prognostic factor for overall survival (OR 0.19, 95% CI 0.06–0.57). Patients who continued previous tyrosine kinase inhibitors (OR 3.48, 95% CI 1.06–11.4) and induced oligoprogression (OR 3.35, 95% CI 1.18–9.52) were associated with worse overall survival. Smoking history (OR 4.27, 95% CI 1.54–11.8) and induced oligoprogression (OR 5.53, 95% CI 2.1–14.7) were associated with worse progression-free survival.

**Conclusions:**

Surgery combined with adjuvant WBRT is a feasible treatment modality for CNS oligoprogression in patients with EGFR-mutated NSCLC. Changing the systemic-targeted therapy after local treatments may be associated with improved overall survival.

**Supplementary Information:**

The online version contains supplementary material available at 10.1186/s12957-023-03248-7.

## Background

Epidermal growth factor receptor (EGFR) mutations account for 10–50% of non-small cell lung cancer (NSCLC) cases, in which a wide range of variation is related to different races [1]. Patients with NSCLC harboring EGFR mutations are prone to central nervous system (CNS) metastasis. Approximately 23–32% of EGFR-mutated NSCLC patients have CNS metastasis at diagnosis, and up to 70% develop CNS metastasis during their treatment course [2, 3]. Patients with oncogene-driven NSCLC have shown better long-term survival in recent decades with advancements in targeted therapy [4, 5]. However, prolonged survival is associated with a risk of systemic disease progression and CNS metastasis after developing tyrosine kinase inhibitor (TKI) resistance [6, 7].

In NSCLC patients with EGFR mutation, disease progression in the CNS is unique to other organs since the CNS has a highly specialized neurovascular structure, the blood–brain barrier (BBB), which limits drug penetration and may serve as a sanctuary for tumor cells [2, 8]. First- and second-generation TKI have limited BBB penetration rates [9]. Therefore, the prevalence of acquired resistance, such as that caused by the T790M mutation, may differ between extracranial and intracranial metastases [10, 11]. Third-generation TKI act against T790M mutations and have adequate BBB penetration rates. Several studies have demonstrated the efficacy of osimertinib in patients with extracranial disease and T790M mutations [12]. However, only a few studies have examined patients with CNS disease progression [13]. The optimal management strategies for such patients can differ from those for extracranial disease progression because newer TKI have the advantage of crossing the BBB.

Oligoprogressive disease is defined as limited sites of treatment failure at the metastatic sites [14, 15]. The management of such patients includes a multidisciplinary approach comprising local ablative therapies, TKI, chemotherapies, and immunotherapies [16–19]. Combining local therapies and TKI can prolong survival compared to TKI alone in patients with EGFR mutation [20–22]. However, patients with CNS oligoprogression have been underrepresented in the existing trials; evidence regarding whether these patients receive the same benefits of aggressive local therapy as other body parts is lacking [19, 23]. In addition, outcomes and optimal subsequent therapies for these patients are not determined. Therefore, this retrospective study aimed to investigate the survival outcomes of surgery with adjuvant whole-brain radiation therapy (WBRT) and subsequent therapies for CNS oligoprogression in EGFR-mutated NSCLC patients.

## Methods

### Patient selection

**Fig. 1:**
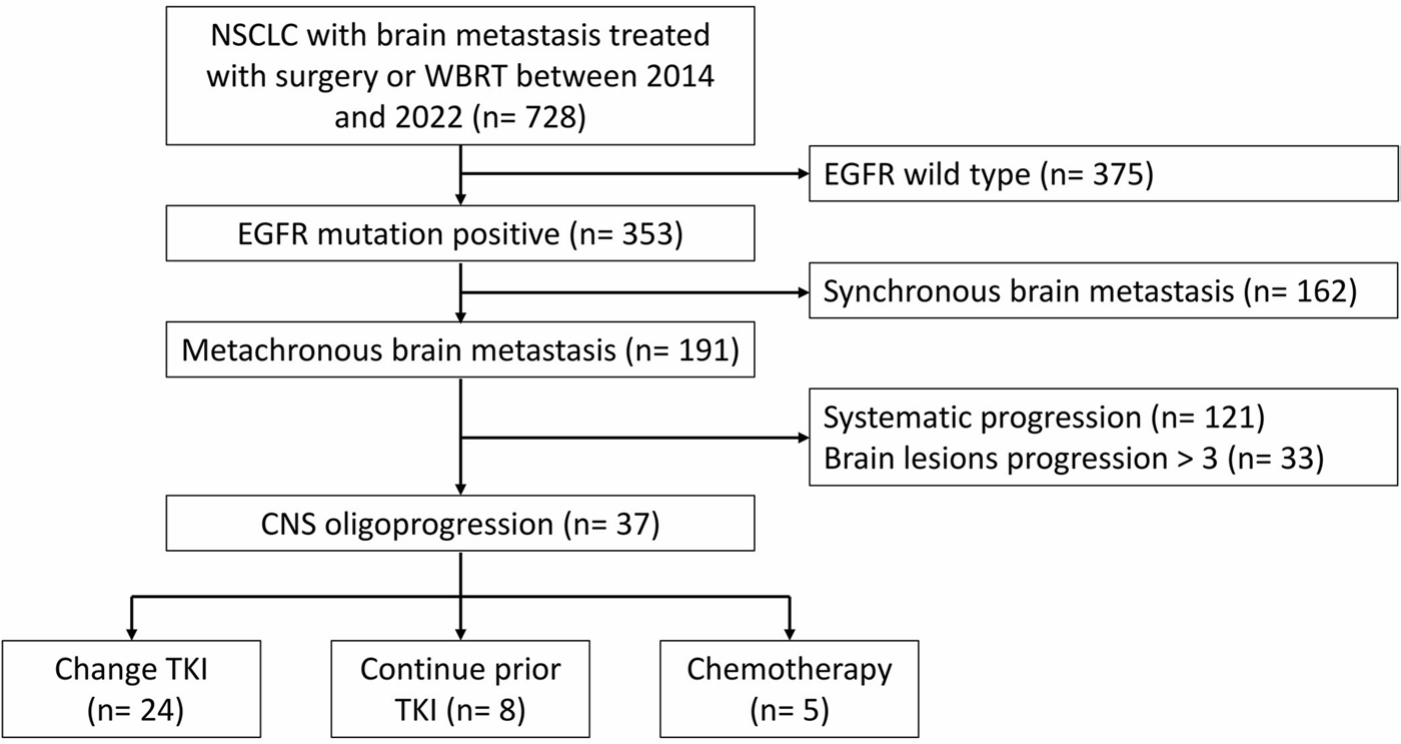
Diaphragm of patient selection. NSCLC, non-small cell lung cancer; EGFR, epidermal growth factor receptor; TKI, tyrosine kinase inhibitors; WBRT, whole brain radiation therapy

All patients with NSCLC diagnosed with CNS metastasis were recruited from a tertiary medical center between 2014 and 2022. The Institutional Review Board of our institute approved this study. Patients who underwent craniotomy tumor excision followed by adjuvant WBRT or WBRT alone for brain metastases were reviewed. CNS oligoprogression was defined as ≤ 3 CNS metastasis increase in size or newly found ≤ 3 CNS metastasis. The definitions of induced oligoprogression, metachronous oligoprogression, and repeat oligoprogression were based on the recent ESTRO/EORTC Oligometastatic Disease Classification [14]. Gadolinium-enhanced brain MRI was performed on each patient before local therapy to evaluate the number and extent of metastases. Imaging studies of chest computed tomography or any extracranial metastatic sites were performed to confirm the diagnosis of oligoprogression in the CNS. Surgical metastasectomy with adjuvant WBRT was indicated for patients with symptomatic or > 3 cm CNS oligoprogression lesions. Subsequent therapy was based on discussions with a multidisciplinary oncology team and whether an exon 20 T790M mutation existed. The diagram used for patient selection is shown in Fig. 1.

### Data collection

The following baseline variables were collected: age, sex, smoking history, Karnofsky performance scale (KPS) score, extracranial metastatic condition, and EGFR mutation subtypes. EGFR mutation subtypes were tested using polymerase chain reaction of primary or metastatic tumor pathology tissues. The T790M plasma test was performed in patients with clinically suspected resistance but with negative tissue pathology results for T790M mutation. Data on tumor-related variables were collected, including tumor size, number of CNS progression lesions, and histopathology. The primary and secondary outcomes were overall survival (OS) and progression-free survival (PFS), respectively. OS and PFS were defined from the date of surgery or WBRT completion until the patient’s death or last follow-up and from the date of disease progression in the primary tumor or any metastatic sites, respectively.

### Statistical analysis

Baseline characteristics were analyzed and presented as medians with interquartile ranges (IQR) or medians with 95% confidence intervals (CI). Kaplan–Meier curves were used to compare OS and PFS. In addition, the log-rank and Breslow tests were used to compare survival differences between the groups for each outcome variable. COX proportional hazards model with univariate and multivariate analyses was used to calculate the odds ratios (ORs) and associated 95% confidence intervals. Variables with a *p*-value less than 0.20 by univariate analysis were included in the multivariate regression. A variance inflation factor value > 2 was considered multicollinear. The results were two-sided; a *p*-value < 0.05 was considered statistically significant. Analysis was performed using SPSS 22 and R software 4.2.2.

## Results

The basic patient demographics are listed in Table 1. Thirty-seven patients were included, with a median survival of 27 months (95% CI 16–37). The median time from diagnosis to the time of CNS oligoprogression was 34 months (95% CI 28–39). All patients had adenocarcinoma pathology. The EGFR mutation subtypes for exon 19 deletion (del 19), exon 21 L858R (L858R), and others (exon 21 L861Q, Exon 18 G719X, and Exon 20 S768I) were 30%, 62%, and 8%, respectively. The remaining tumor-related characteristics are listed in Table 1.

**Table 1:** General demographic

		*N* = 37	%
Age, median (IQR)		61 (58–68)	
Male		13	35
Smoking history		6	16
Karnofsky performance scale	> 80	22	59
	70–80	13	35
	< 70	2	5
Initial stage	I	5	14
	II	2	5
	III	13	35
	IV	17	46
EGFR mutation	Del 19	11	30
	L858R	23	62
	Others	3	8
Pathology	Adenocarcinoma	37	100
Tumor number	1	13	35
	2–3	24	65
Main tumor location	Supratentorial	30	81
	Infratentorial	7	19
Treatment	Surgery	21	57
	WBRT	16	43
Subsequent therapy	Change TKI	24	65
	Keep TKI	8	22
	Chemotherapy	5	14
Oligoprogression type	Repeat or Metachronous	17	46
	Induced	20	54

*Del 19*, exon 19 deletion; *L858R*, exon 21 L858R; *Others*, Exon 21 L861Q, Exon 18 G719X, and Exon 20 S768I; *IQR*, interquartile range; *TKI*, tyrosine kinase inhibitors; *WBRT*, whole brain radiation therapy

Seventeen patients had metachronous or repeated CNS oligoprogression, whereas the others had induced CNS oligoprogression. Twenty-four (65%) patients changed their TKI after local therapies for CNS oligoprogression, while 8 (22%) patients continued their previous TKI, and 5 (14%) patients changed to chemotherapy. In the patient group who changed their TKI treatment, 13 (54%) patients switched to osimertinib. The T790M mutation was subsequently detected in 7 (19%) among these 13 patients, whereas the rest tested negative for the mutation. Eleven patients who changed TKI were T790M-negative (two patients shifted from afatinib to erlotinib, one from erlotinib to afatinib, two from gefitinib to afatinib, and six from gefitinib to erlotinib).

**Fig. 2:**
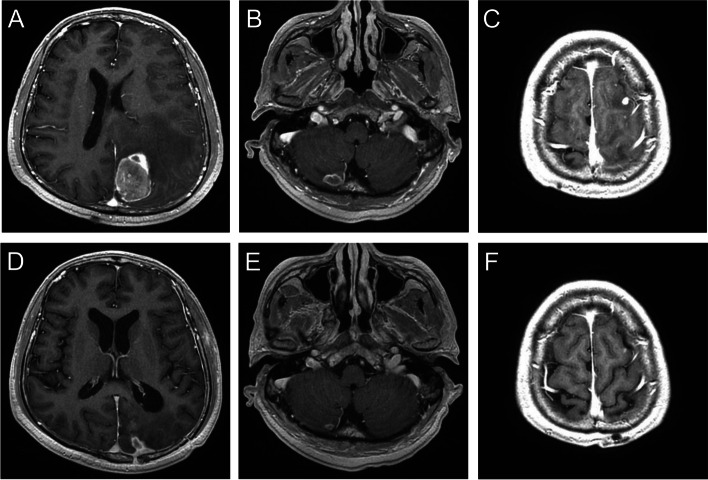
T1-weighted gadolinium-enhanced brain MRI of a 58-year-old man. Oligoprogression lesions were found in the left occipital (**A**), left frontal lobe (**B**), and right cerebellum (**C**). After left occipital tumor excision and adjuvant whole-brain radiotherapy, the patient changed from afatinib to osimertinib. Lesions were controlled well after a 6-month image follow-up (**D**, **E**, **F**)

**Fig. 3:**
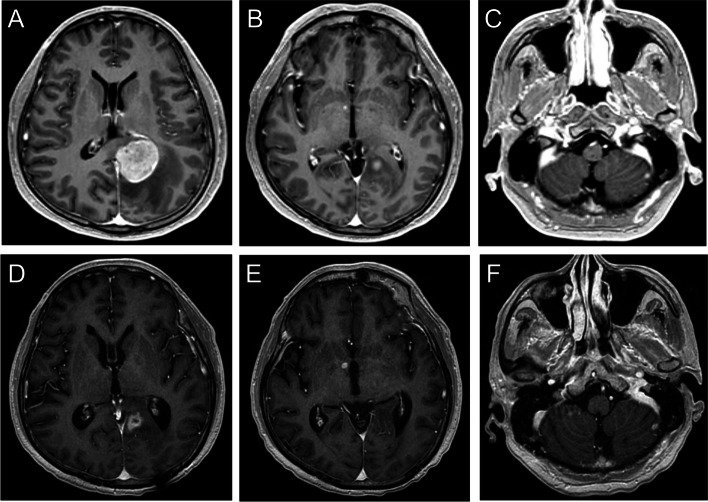
T1-weighted gadolinium-enhanced brain MRI of a 64-year-old man. Olioprogression lesions were noted in left occipital lobe (**A**), right basal ganglia (**B**), and left cerebellum (**C**). The patient continued afatinib after surgically removing the left occipital tumor with adjuvant WBRT. After a 6-month follow-up, the tumor was controlled in the left occipital (**D**) but progressed in the right basal ganglia (**E**) and left cerebellum (**F**)

Two illustrative cases that changed TKI or continued previous TKI therapy are presented in Figs. 2 and 3, respectively. A 58-year-old man was diagnosed with stage IIIB NSCLC in 2014. Afatinib treatment was initiated. Subsequently, in 2019, the patient developed CNS oligoprogression in the left frontal lobe, left occipital lobe, and right cerebellum (Figs. 2A, B, C). He underwent left occipital metastasectomy with adjuvant WBRT in September 2019. Osimertinib was administered despite negative results for the T790M mutation in the excised brain tissue. A follow-up MRI performed in January 2020 showed regression of all lesions in the CNS (Figs. 2D, E, and F). The second patient was a 64-year-old man diagnosed with initial stage IV NSCLC owing to bone metastasis. Since then, afatinib has been administered. CNS oligoprogression was detected in the left occipital lobe, right basal ganglia, and left cerebellum in January 2018 (Figs. 3A, B, and C). After surgical removal of the main tumor in the left occipital lobe, followed by WBRT, the patient tested negative for the T790M mutation in tissue pathology and plasma circulation DNA. Therefore, the patient continued the TKI treatment. However, the six monthly follow-up brain MRI showed progression of all existing brain lesions except for the excised lesion (Figs. 3D, E, F).

### Overall survival analysis

**Fig. 4:**
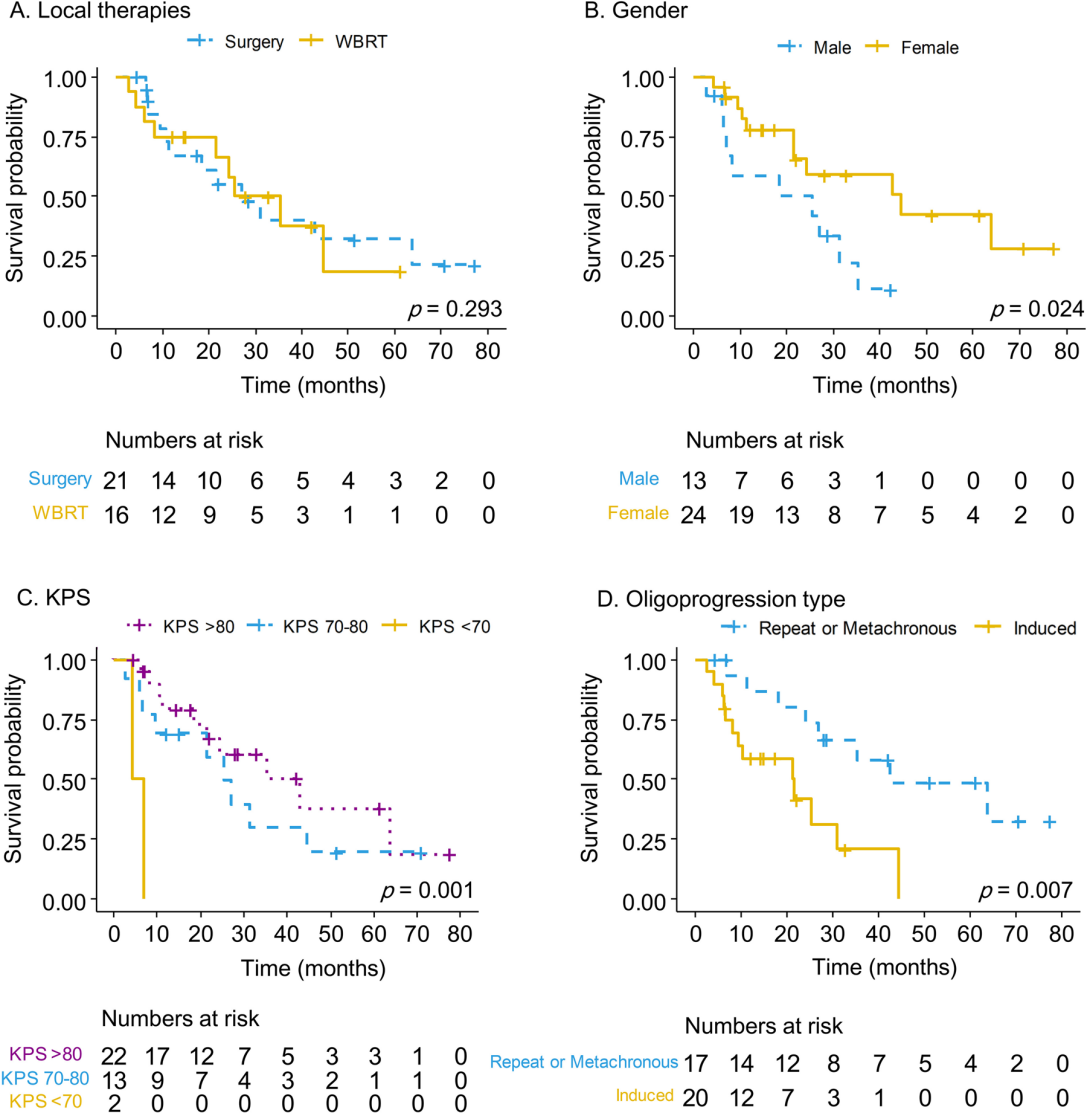
The Kaplan–Meier survival curve for different variables, including local therapies (**A**), genders (**B**), Karnofsky performance scale (KPS) (**C**), and different oligoprogression types (**D**) in patients with NSCLC central nervous system oligoprogression

**Fig. 5:**
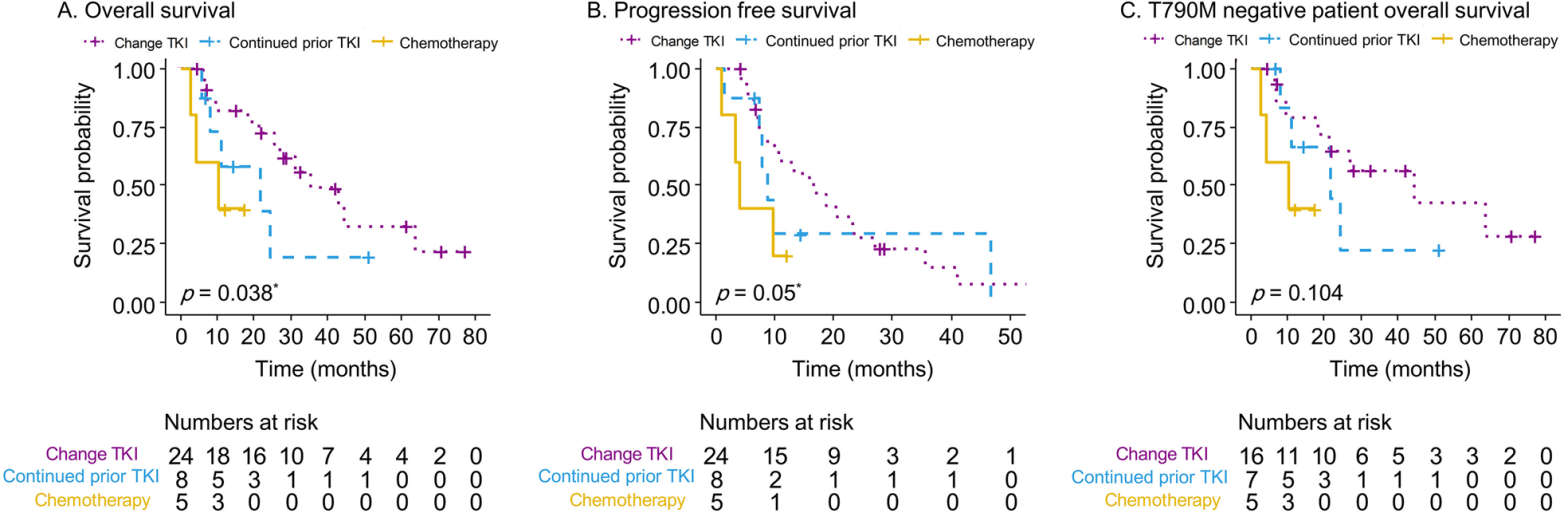
The Kaplan–Meier survival curve of overall survival (**A**), progression-free survival (**B**), and overall survival for T790M negative patients who received different subsequent therapies (**C**) in NSCLC central nervous system oligoprogessive disease. *p value done by Breslow method

The median survival of patients who underwent surgery with adjuvant WBRT and WBRT alone were 43 (95% CI 17–69) and 22 (95% CI 15–29) months, respectively. No statistically significant differences were noted among the different local therapies (Fig. 4). Female sex, metachronous or repeat oligoprogression types, patients who changed their TKI, and KPS > 70 were associated with a better OS (Fig. 4, Fig. 5A, and Table S1). For T790M-negative patients, the median survival for different subsequent therapies was 61 (95% CI 19–102), 22 (95% CI 0–44), and 11 (95% CI 0–26) months with the *p*-value of 0.104 (Fig. 5C). In multivariate analysis, female sex (OR 0.19, 95% CI 0.06–0.57) was associated with better OS. However, patients who continued their previous TKI (OR 3.48, 95% CI 1.06–11.4) and induced oligoprogression type (OR 3.35, 95% CI 1.18–9.52) were associated with a worse OS (Table 2).

**Table 2:** Univariate and multivariate analysis for overall survival

			Univariate				Multivariate			
		*N*	OR	95% CI		*p* value	OR	95% CI		*p* value
				Lower	Upper			Lower	Upper	
Age		37	0.99	0.95	1.032	0.646				
Female		24	0.36	0.141	0.916	0.032	0.19	0.063	0.574	0.003
Smoking history		6	1.285	0.422	3.917	0.659				
KPS		37	1.006	0.99	1.024	0.452				
Extracranial metastasis present		16	1.08	0.446	2.617	0.865				
EGFR mutation	19 del	11								
	L858R	23	0.842	0.315	2.248	0.732				
	Others	3	1.564	0.304	8.046	0.593				
Tumor number	1	13								
	2–3	24	0.811	0.339	1.943	0.639				
Main tumor location	Supratentorial	30								
	Infratentorial	7	1.943	0.704	5.368	0.2				
Main tumor size		37	1.007	0.764	1.327	0.96				
Treatment	Surgery	21								
	WBRT	16	1.602	0.658	3.9	0.299				
Subsequent therapy	Change TKI	24								
	Keep TKI	8	2.095	0.722	6.078	0.174	3.476	1.057	11.429	0.04
	Chemotherapy	5	4.527	1.097	18.672	0.037	6.988	1.286	37.962	0.024
Oligoprogression type	Repeat or Metachronous	17								
	Induced	20	3.525	1.335	9.306	0.011	3.346	1.177	9.517	0.024

*Del 19*, exon 19 deletion; *KPS*, Karnofsky performance scale; *L858R*, exon 21 L858R; *Others*, Exon 21 L861Q, Exon 18 G719X, and Exon 20 S768I; *TKI*, tyrosine kinase inhibitors; *WBRT*, whole brain radiation therapy

### Progression-free survival analysis

The overall PFS was 11 (95% CI 4.7–17) months. The log-rank test demonstrated that metachronous or repeat oligoprogression types, non-smoking status, and KPS > 70 were associated with better PFS (Table S1). In contrast, subsequent therapies showed a borderline statistically significant difference (*p* = 0.05) (Fig. 5B). Multivariate analysis revealed that smoking history (OR 4.27, 95% CI 1.54–11.8) and induced oligoprogression (OR 5.53, 95% CI 2.1–14.7) were associated with worse PFS, while female sex (OR 0.43, 95% CI 0.06–0.57) was not (Table S2).

## Discussion

This study evaluated surgical outcomes with subsequent therapies for EGFR-mutated NSCLC patients with CNS oligoprogression. The median survival after surgery with adjuvant WBRT and WBRT alone were 43 (95% CI 17–69) and 22 (95% CI 15–29) months, respectively. The positive prognostic factors for OS were female sex and metachronous or repeat oligoprogression. Furthermore, we noted that patients who subsequently switched to another systemic TKI had a better OS than those who continued previous TKI therapy or switched to chemotherapy.

Optimal treatment guidelines in clinical practice are under investigation despite skyrocketing discussions on oligoprogression [21]. Stereotactic body radiation therapy can prolong median survival to 28–37 months and PFS to 7–10 months for oligoprogressive EGFR-mutated NSCLC without CNS involvement [24, 25]. However, surgical outcomes for patients with oligoprogression have rarely been reported, especially in the CNS [26, 27]. In our cohort, we analyzed OS and PFS, specifically focusing on patients with CNS oligoprogression. The outcomes were comparable to those of non-CNS oligoprogression in NSCLC patients treated with radiation therapy [22, 24, 25] or surgery [28]. In contrast to stereotactic radiotherapy, surgery has the advantages of providing a proven diagnosis, tumor genomic testing, and immediate relief from the mass effect. We provided the rationale for craniotomy, and adjuvant WBRT could be a treatment modality for EGFR-mutated CNS oligoprogression despite the limited number of cases, which may lead to a non-significantly superior survival compared to WBRT alone.

Oligoprogressive diseases can be classified into induced, metachronous, and repeat types based on the patient’s underlying condition [14, 26]. In our analysis, the induced type had much worse outcomes than the metachronous or repeat type, which is consistent with a previous study focusing on the survival outcomes of stereotactic body radiation therapy for oligometastatic and oligoprogressive lesions [29]. These lesions are mainly in the lungs, bones, and liver. Likewise, a study by Baker et al. using the data from the Stereotactic Ablative Body Radiotherapy (SABR) 5 Trial for the prognostication of different oligometastatic and oligoprogression types exhibited similar results [30]. Furthermore, a study that included patients with NSCLC who had extracranial oligoprogression treated with definitive radical radiotherapy concluded that induced oligoprogression was associated with worse survival [31]. However, several earlier studies reporting the survival and PFS outcomes of stereotactic body radiation therapy for NSCLC oligoprogression did not identify induced oligoprogression as a prognostic factor for OS [24, 25, 32]. A possible explanation is that the newer oligoprogressive and oligometastatic disease classifications were not clearly defined until an expert consensus was reached in 2020. The proposed mechanisms for the worse outcomes of induced oligoprogression were more aggressive cancer patterns [28]. Our PFS analysis supports this hypothesis, since these induced-type patients had shorter PFS, indicating a poor response to the original or new TKI treatment.

Our analysis demonstrates that shifting to a new TKI exerts a survival benefit in patients with CNS oligoprogression after local therapies. Controversies exist regarding whether to continue previous TKI therapy or change TKI when combining local therapies for patients with oligoprogressive EGFR-mutated NSCLC. Although several pilot studies have shown that continuing first-line TKI has survival benefits and could preserve second- or third-line therapies upon future disease progression, our analysis revealed otherwise [20, 27, 32, 33]. Several factors may have contributed to this finding. First, the shift from ongoing TKI to osimertinib can address T790M-related resistance. Although only 19% of patients developed T790M mutation in this cohort, consistent with previous studies [10], the actual rate of T790M mutation may be higher given the limited accuracy of circulating tumor DNA analysis data for patients with CNS progression [34]. Second, because osimertinib exhibits better CNS penetration, it is more effective in patients with EGFR-mutated NSCLC brain metastasis treated with previous first- and second-generation TKI [35]. This may explain the trend of survival benefits when osimertinib was administered to T790M-negative patients (Fig. 5C) [36]. Third, the gene mutation discordance in NSCLC between primary and brain metastases has been reported to be up to 10% [37, 38]. In addition, genomic sequencing studies have demonstrated that CNS metastases from NSCLC show higher amplification in regions such as *MYC* and *YAP* compared to primary lung lesions [39]. Therefore, CNS oligoprogression may behave differently in terms of nature and activity than extracranial metastasis [33]. Continued TKI therapy may lead to insufficient control of tumor progression [40]. In line with the illustrative cases, we demonstrated that the first patient who changed TKI therapy had better tumor control for non-target lesions. Contrarily, the non-excised lesions in case two showed disease progression despite using the same TKI. However, in our analysis, PFS did not differ significantly between subsequent therapies (Fig. 5B). Further high-level evidence-based studies are required to confirm this concept.

In summary, NSCLC patients with CNS oligoprogression harboring EGFR mutations constitute a distinct subgroup warranting individualized treatment plans before therapy initiation. Local ablative therapies hold promise for managing extracranial oligoprogression; however, optimal patient selection for local therapies in CNS oligoprogression remains unclear. Likewise, the efficacy of upfront versus adjuvant local therapies and identifying the most suitable approach for individual patients, including surgery, stereotactic body radiation therapy, and other ablation techniques such as thermotherapies, are ongoing areas of investigation in this context. Several prospective trials are underway to provide higher-level evidence for managing oligoprogressive disease in NSCLC patients [41]. Novel therapies targeting EGFR resistance have emerged, with tissue next-generation sequencing and circulating DNA analysis aiding in identifying potential targetable genes in these patients [42]. A deeper understanding of resistance mechanisms may improve overall survival in NSCLC patients with oligoprogression and oligometastatic disease. Tissue next-generation sequencing plays a vital role in exploring intrinsic resistance and tumor adaptation pathways, further supporting the role of surgery for treatment in oligoprogression patients.

This study has some limitations. The first is the limited number of cases and the nature of a single institution, and several confounding factors were present in this retrospective study. The complicated underlying conditions of such metachronous patients require prospective trials to prove our concept despite the multivariate analysis. Second, T790M detection was based on the polymerase chain reaction results of the excised tissues in patients who underwent surgical craniotomy or plasma samples from patients who received radiotherapy alone. Circulating tumor DNA cannot detect definite T790M mutation status, especially in patients with isolated CNS progression [34]. Third, although promising results have been proposed for stereotactic radiosurgery in treating tumor beds after surgical resection for CNS metastatic lesions, this procedure has not yet become a routine protocol in our national insurance system. Therefore, the efficacy of stereotactic radiosurgery in these patients remains unclear. Fourth, this research did not discuss patients receiving novel therapies, including immunotherapies and new MET-targeting agents such as capmatinib, due to the limited case numbers.

## Conclusion

The optimal treatment strategy for CNS oligoprogression in patients with EGFR-mutated NSCLC remains unknown. Despite the limited number of cases, our results support using surgical resection with adjuvant WBRT as a treatment modality for such patients. Changing systemic targeted therapy may be associated with a survival benefit compared to continuing previous targeted therapy or chemotherapy after local treatments.

### Supplementary Information

**Additional file 1.** 

## Data Availability

The data that support the findings of this study are available on request from the corresponding author. The data are not publicly available due to privacy or ethical restrictions.

## Abbreviations

BBB: Blood–brain barrier
CI: Confidence interval
CNS: Central nervous system
EGFR: Epidermal growth factor receptor
IQR: Interquartile ranges
KPS: Karnofsky performance scale
NSCLC: Non-small cell lung cancer
OS: Overall survival
PFS: Progression-free survival
TKI: Tyrosine kinase inhibitor
WBRT: Whole brain radiation therapy
