# Supplementary material for: Outcomes of surgery and subsequent therapy for central nervous system oligoprogression in EGFR-mutated NSCLC patients
Source: World J Surg Oncol. 2023 Nov 25;21:368. doi: 10.1186/s12957-023-03248-7 (PMC10675964; doi:10.1186/s12957-023-03248-7)
Supplement: Supplementary file 1 — Additional file 1. [file 12957_2023_3248_MOESM1_ESM.docx]

| Table S1 Kaplan-Meier curve analysis for medain survival of different variables | | | | |  |  |  |
| --- | --- | --- | --- | --- | --- | --- | --- |
|  |  | Overall Survival | | | Progression free survival | | |
|  |  | Median | 95% CI | p value | Median | 95% CI | p value |
| Sex | Male | 26 | 0-56 | 0.024 | 10 | 3-17 | 0.105 |
|  | Female | 44 | 14-74 |  | 16 | 1-31 |  |
| Smoking history | Yes | 35 | 0-92 | 0.656 | 5 | 0-13 | 0.037 |
|  | No | 27 | 18-36 |  | 16 | 10-22 |  |
| KPS | >70 | 43 | 19-67 | 0.001 | 13 | 5-21 | 0.011 |
|  | 70-80 | 26 | 17-35 |  | 10 | 0-23 |  |
|  | <70 | 4 | NA |  | 4 | NA |  |
| Extracranial metastasis | Yes | 26 | 12-40 | 0.864 | 10 | 6-14 | 0.153 |
|  | No | 27 | 13-41 |  | 17 | 5-29 |  |
| EGFR mutation | Del 19 | 26 | 0-57 | 0.711 | 10 | 6-14 | 0.743 |
|  | L858R | 31 | 19-43 |  | 16 | 4-27 |  |
|  | Others | 22 | NA |  | 9 | NA |  |
| Tumor number | 1 | 27 | 8-46 | 0.637 | 17 | 0-40 | 0.705 |
|  | 2-3 | 31 | 19-43 |  | 10 | 8-12 |  |
| Main tumor location | Supratentorial | 35 | 20-50 | 0.19 | 11 | 7-15 | 0.341 |
|  | Infratentorial | 21 | 0-47 |  | 19 | 7-31 |  |
| Treatment | Surgery | 43 | 17-69 | 0.293 | 16 | 8-24 | 0.127 |
|  | WBRT | 22 | 15-29 |  | 8 | 6-10 |  |
| Subsequent therapy | Change TKI | 35 | 17-53 | 0.038* | 17 | 8-26 | 0.05* |
|  | Keep TKI | 22 | 0-44 |  | 9 | 6-12 |  |
|  | Chemotherapy | 11 | 0-26 |  | 4 | 1-7 |  |
| Oligoprogression type | Repeat or Metachronous | 43 | 12-74 | 0.007 | 23 | 5-41 | 0.003 |
|  | Induced | 22 | 5-39 |  | 8 | 5-11 |  |
| Del 19: exon 19 deletion; L858R: exon 21 L858R; Others: Exon 21 L861Q, Exon 18 G719X, and Exon 20 S768I; TKI: tyrosine kinase inhibitors; WBRT: whole brain radiation therapy | | | | | | | |
| * Analyzed with Breslow test | |  |  |  |  |  |  |

| Table S2. Univariate and Multivariate analysis for progression free survival | | | |  |  |  |  |  |  |  |
| --- | --- | --- | --- | --- | --- | --- | --- | --- | --- | --- |
|  |  |  | Univariate | | | | Mulivariate | | | |
|  |  | N | OR | 95% CI | | p value | OR | 95% CI | | p value |
|  |  |  |  | Lower | Upper |  |  | Lower | Upper |  |
| Age |  | 37 | 0.973 | 0.938 | 1.009 | 0.138 |  |  |  |  |
| Female |  | 24 | 0.538 | 0.246 | 1.175 | 0.12 | 0.429 | 0.19 | 0.966 | 0.041 |
| Smoking history |  | 6 | 2.511 | 1 | 6.309 | 0.05 | 4.271 | 1.54 | 11.839 | 0.005 |
| KPS |  | 37 | 1.009 | 0.994 | 1.024 | 0.247 |  |  |  |  |
| Extracranial metastasis present | | 16 | 1.735 | 0.791 | 3.804 | 0.169 |  |  |  |  |
| EGFR mutation | 19 del | 11 |  |  |  |  |  |  |  |  |
|  | L858R | 23 | 0.783 | 0.35 | 1.753 | 0.552 |  |  |  |  |
|  | Others | 3 | 1.198 | 0.248 | 5.778 | 0.822 |  |  |  |  |
| Tumor number | 1 | 13 |  |  |  |  |  |  |  |  |
|  | 2-3 | 24 | 1.159 | 0.529 | 2.54 | 0.712 |  |  |  |  |
| Main tumor location | Supratentorial | 30 |  |  |  |  |  |  |  |  |
|  | Infratentorial | 7 | 1.6 | 0.589 | 4.345 | 0.357 |  |  |  |  |
| Main tumor size |  | 37 | 0.958 | 0.752 | 1.219 | 0.958 |  |  |  |  |
| Treatment | Surgery | 21 |  |  |  |  |  |  |  |  |
|  | WBRT | 16 | 0.566 | 0.265 | 1.209 | 0.142 |  |  |  |  |
| Subsequent therapy | Change TKI | 24 |  |  |  |  |  |  |  |  |
|  | Keep TKI | 8 | 1.292 | 0.502 | 3.323 | 0.595 |  |  |  |  |
|  | Chemotherapy | 5 | 3.025 | 0.962 | 9.515 | 0.058 |  |  |  |  |
| Oligoprogression type | Repeat or Metachronous | 17 |  |  |  |  |  |  |  |  |
|  | Induced | 20 | 3.506 | 1.452 | 8.464 | 0.005 | 5.533 | 2.09 | 14.651 | 0.001 |
| Del 19: exon 19 deletion; L858R: exon 21 L858R; Others: Exon 21 L861Q, Exon 18 G719X, and Exon 20 S768I; TKI: tyrosine kinase inhibitors; WBRT: whole brain radiation therapy | | | | | | | | | | |
